# Production and Evaluation of a Recombinant Chimeric Vaccine against *Clostridium botulinum* Neurotoxin Types C and D

**DOI:** 10.1371/journal.pone.0069692

**Published:** 2013-07-31

**Authors:** Luciana A. F. Gil, Carlos Eduardo P. da Cunha, Gustavo M. S. G. Moreira, Felipe M. Salvarani, Ronnie A. Assis, Francisco Carlos F. Lobato, Marcelo Mendonça, Odir A. Dellagostin, Fabricio R. Conceição

**Affiliations:** 1 Faculdade de Veterinária, Universidade Federal de Pelotas, Pelotas, RS, Brazil; 2 Centro de Desenvolvimento Tecnológico, Núcleo de Biotecnologia, Universidade Federal de Pelotas, Pelotas, RS, Brazil; 3 Escola de Veterinária, Universidade Federal de Minas Gerais, Belo Horizonte, MG, Brazil; Institute Pasteur, France

## Abstract

Bovine botulism is a fatal disease that is caused by botulinum neurotoxins (BoNTs) produced by *Clostridium botulinum* serotypes C and D and that causes great economic losses, with nearly 100% lethality during outbreaks. It has also been considered a potential source of human food-borne illness in many countries. Vaccination has been reported to be the most effective way to control bovine botulism. However, the commercially available toxoid-based vaccines are difficult and hazardous to produce. Neutralizing antibodies targeted against the C-terminal fragment of the BoNT heavy chain (H_C_) are known to confer efficient protection against lethal doses of BoNTs. In this study, a novel recombinant chimera, consisting of *Escherichia coli* heat-labile enterotoxin B subunit (LTB), a strong adjuvant of the humoral immune response, fused to the H_C_ of BoNT serotypes C and D, was produced in *E. coli*. Mice vaccinated with the chimera containing LTB and an equivalent molar ratio of the chimera without LTB plus aluminum hydroxide (Al(OH)_3_) developed 2 IU/mL of antitoxins for both serotypes. Guinea pigs immunized with the recombinant chimera with LTB plus Al(OH)_3_ developed a protective immune response against both BoNT/C (5 IU/mL) and BoNT/D (10 IU/mL), as determined by a mouse neutralization bioassay with pooled sera. The results achieved with guinea pig sera fulfilled the requirements of commercial vaccines for prevention of botulism, as determined by the Brazilian Ministry of Agriculture, Livestock and Food, Supply. The presence of LTB was essential for the development of a strong humoral immune response, as it acted in synergism with Al(OH)_3_. Thus, the vaccine described in this study is a strong candidate for the control of botulism in cattle.

## Introduction

Botulism is a severe and fatal disease characterized by flaccid paralysis due to the inhibition of acetylcholine release at the neuromuscular junction caused by the botulinum neurotoxins (BoNTs) produced by *Clostridium* *barati* *,* *C* *. butyricum* *,* *C* *. argentinense* and, most commonly, by *C. botulinum* [1], a Gram-positive anaerobe rod and spore-forming bacteria present in soil, water and decaying organic matter. There are seven serotypes of BoNTs (A-G) that vary antigenically, though they have the same pharmacological activity [2].

In many countries, including Brazil, BoNTs serotypes C and D are responsible for causing botulism in cattle [3–5]. Cattle with calcium and phosphorus deficiencies often resort to bone chewing to supplement their lack of minerals, which is the main cause of endemic botulism [6]. Dutra et al. [7] reported seven outbreaks of bovine botulism in Brazil, which were associated with contaminated water and resulted in 99.92% lethality. In another study [8], the same authors reported more than seven outbreaks of bovine botulism due to contaminated bedding for poultry, resulting in 3,299 dead animals. More recently, Costa et al. [5] reported an outbreak of bovine botulism caused by serotypes C and D present in contaminated food, with 100% lethality in a dairy-producing property. Outbreaks of botulism have also been reported in Europe [9,10] and North America [11], where there is also great concern that infected animals will become a source of food-borne botulism for humans. Therefore, this disease is one of the main causes of cattle death and, consequently, of great economic losses; in addition, it is a matter of concern for public safety worldwide.

Vaccination is the most effective method to prevent death via BoNT poisoning [12,13]. Current commercial vaccines are produced by inactivated native toxins (toxoids) combined with conventional adjuvants, which, although efficient, present some production limitations: (1) the amount of toxin produced *in vitro* is unpredictable, and (2) BoNTs are the most potent biological toxins known in nature [14], and therefore high levels of biosafety are required [13]. Thus, it is critical to develop new strategies that can overcome these problems.

BoNTs are synthesized as single polypeptide chain prototoxins and are activated by enzymatic cleavage by *C. botulinum* proteases [1]. The activated form of the toxins is composed of two chains and three characteristic domains. The light chain is a Zn^2+^-metalloprotease, which is connected to the N-terminal half of the heavy chain domain (translocation domain, H_N_) by a single disulfide bond [15]. The C-terminal region of the heavy chain (i.e., the binding domain to the neuronal receptor, H_C_) comprises the region that interacts with neurons. Additionally, the C-terminal region of the heavy chain is a nontoxic domain that possesses protective epitopes [16]. It has already been shown that subunit vaccines made of recombinant H_C_, produced in either *Escherichia coli* or *Pichia* *pastoris* , are capable of inducing neutralizing antibodies and protection against botulism [12,17–21]. Consequently, recombinant subunit vaccines are interesting strategies to solve the problems inherent to toxoid vaccine production.

Chimeric proteins carrying epitopes from different pathogens, linkers, or adjuvant sequences offer increased immunogenicity for recombinant antigens and can also elicit broad immune responses [22–24]. In this study, using only one fermentative and downstream process, we developed a chimeric vaccine, consisting of the H_C_ region of BoNTs serotypes C and D fused to the B subunit of the *Escherichia coli* heat-labile enterotoxin (LTB), a potent adjuvant of the humoral immune response [25,26]. We then further evaluated the immunogenicity of this vaccine in animal models.

## Material and Methods

### Ethics statement

This study was carried out in strict accordance with the recommendations of the Conselho Nacional de Controle de Experimentação Animal (CONCEA). The protocol was approved by the Committee on the Ethics of Animal Experiments of the Federal University of Pelotas (Permit No. 9286). All efforts were made to minimize animal suffering.

### Native botulinum neurotoxins and standard sera against botulinum neurotoxins serotypes C and D

Native botulinum neurotoxins were produced by *Clostridium botulinum* serotypes C strain Onderstepoort Veterinary Institute 01/1992 and D strain Onderstepoort Veterinary Institute 02/1992 obtained from the Onderstepoort Veterinary Institute (South Africa) standardized to 1 L+/mL. Standard sera against BoNTs serotypes C and D were obtained from the Center for Diseases Control (CDC) lot 76.0342 catalog number BS0612 and lot 76.0338 catalog number BS0611, respectively.

### Gene synthesis and cloning

**Figure 1:**
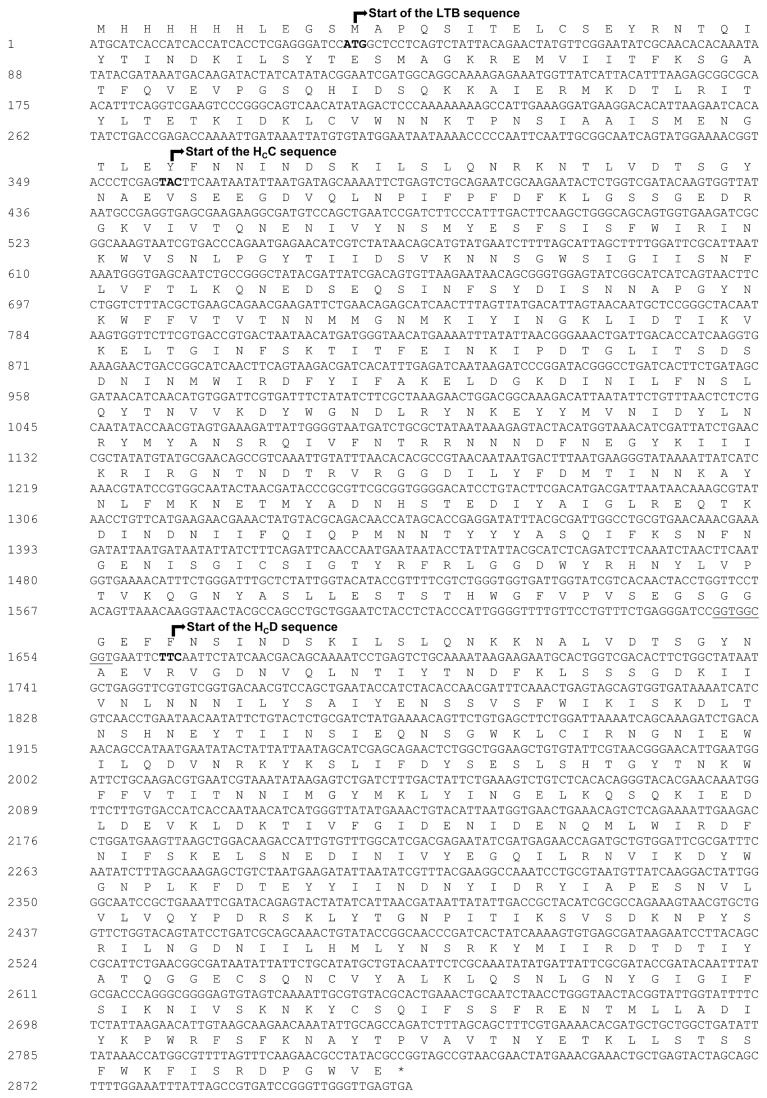
Gene sequence and translation of the synthesized *ltb/h*_*c*_ *c/h*_*c*_ *d* construct. Representation of the synthetic gene which encodes rLTB/H _C_C/H _C_D protein. The first codon of each subunit is in bold and identified by an **arrow**. The 3xGly linker codons are underlined. Stop codon is indicated by an asterisk (*).

A synthetic gene encoding the fused H_C_ of BoNT serotypes C and D (H_C_C/H _C_D) was synthesized by Epoch Biolabs, Inc. (USA) with optimal codon usage for *E. coli*. The protein sequences of H_C_C (gi:115185) and H_C_D (gi:115188) were used as reference to design the gene. Restriction sites were added to allow cloning into the pAE and pAE/*ltb* expression vectors [25] for *E. coli* (Table 1). A three-glycine linker (3xGly) was added between the two H_C_s to enable the proper folding of each H_C_. DNA manipulation was performed according to the protocols previously described by Sambrook and Russel [27]. Briefly, after digestion, electrophoresis on an agarose gel was performed. Bands representing the fragments encoding proteins of interest and expression vectors were purified from the gel or digestion reactions, respectively, using the Gel Band Purification Kit (GE Healthcare) and illustra GFX PCR DNA. After purification, the inserts and vectors were quantified and ligated with T4 DNA ligase (New England Biolabs). The ligation products were used to transform *E. coli* TOP10, which were cultured overnight on LB agar plates with 100 µg/mL ampicillin. Bacterial clones were screened for recombinants. Plasmid DNA was extracted using the alkaline lysis method [27], and recombinant clones were characterized by endonuclease digestion. The gene sequence with optimized codons and its translation are represented in Figure 1.

**Table 1:** Specifications regarding restriction enzymes, gene fragments and chimera characteristics.

Recombinant chimera	Restriction enzymes^a^	Expression vector	Insert	Fragment length (pb)	Molecular mass (kDa)^b^
rLTB/H_C_C/H_C_D	*Xho*I and *Hin*dIII	pAE/*ltb*	*h*_*c*_ *c/h*_*c*_ *d*	2562	112.4
rH_C_C/H_C_D	*Kpn*I and *Hin*dIII	pAE	*h*_*c*_ *c/h*_*c*_ *d*	2568	100.0

^a^ Purchased from New England Biolabs

^b^ Predicted by Vector NTI Advance 11 (Invitrogen)

### Expression and purification of the recombinant chimeras

The recombinant vectors were transformed into *E. coli* BL21 (DE3) Star by heat shock method [27]. Bacteria transformed with each recombinant vector were grown in Luria-Bertani (LB) broth supplemented with 100 µg/mL of ampicillin in a shaker (37 °C, 150 RPM) until the mid-log growth phase (OD_600_=0.6-0.8). Heterologous protein expression was induced for 3 h by the addition of isopropyl β-D-1-thiogalactopyranoside (IPTG) to a final concentration of 0.5 mM. The cells were harvested by centrifugation (16,000 *x g*, 10 min, 4 °C), suspended in lysis buffer (0.2 M NaH_2_PO_4_, 0.5 M NaCl, 10 mM imidazole), incubated with 50 µg/mL of lysozyme for 1 h at room temperature, disrupted by sonication and centrifuged again (10,000 *x g*, 30 min, 4 °C). The pellets with inclusion bodies were washed three times with lysis buffer and incubated overnight at 4 °C with lysis buffer supplemented with 0.2% or 0.4% (w/v) N-lauroylsarcosine (NLS) and centrifuged again (10,000 *x g*, 30 min, 4 °C). The recombinant proteins were purified by affinity chromatography, using HisTrap^TM^ HP 1 ml columns Ni Sepharose^TM^ on the ÄKTAprime^TM^ automated liquid chromatography system (GE Healthcare), according to the manufacturer’s instructions. Purified fractions were dialyzed against phosphate buffered saline (PBS, pH 7.4) with 0.05% (v/v) Triton X-100 overnight and lyophilized until further use. Protein quantification was performed using the BCA^TM^ Protein Assay (Pierce). The purity and integrity of the recombinant antigens were evaluated by 12% SDS-PAGE.

### Characterization of the recombinant chimeras

Approximately 5 µg of each of the purified proteins were run in SDS-PAGE 12% and then transferred to a Hybond-ECL nitrocellulose membrane (GE Healthcare) using Tris-Glycine transfer buffer (Tris 25 mM, Glycine 0.2 M, methanol 20% [v/v]) for 1 h under 100 V and approximately 400 mA. The membrane was blocked for 1 h at room temperature with non-fat dry milk 5% (w/v) diluted in PBS with 0.05% (v/v) Tween 20 (PBS-T). The membrane was incubated with mouse monoclonal anti-polyHistidine antibody (Sigma-Aldrich) diluted 1:6,000 in PBS-T for 1 h at room temperature. Rabbit anti-mouse IgG antibody conjugated with peroxidase (Sigma Aldrich) was diluted 1:4,000 in PBS-T and used as secondary antibody. The membrane was incubated the same way as before and revealed by chemiluminescent assay using ECL Western blotting substrate (Thermo Scientific) according to manufacturer’s instructions. After each incubation step, three washes were performed with PBS-T in order to eliminate residual reagents.

The ELISA was performed to evaluate the antigenicity of the chimeras. At each step, plates were incubated for 1 h at 37 °C. Flat-bottom 96-well plates (Nunc) were coated with 200 ng per well of rLTB/H_C_C/H _C_D diluted in coating buffer (carbonate-bicarbonate 0.2 M, pH 9.7). The other antigens were added at the same molar ratio. Plates were blocked with non-fat dry milk 5% (w/v) diluted in PBS-T. The standardized anti-toxin C or *D* *sera* were diluted in PBS-T to 1 IU/mL and rabbit anti-cholera toxin antibody (Sigma Aldrich) was diluted 1:4,000 before they were applied to the plates. Rabbit anti-sheep IgG (1:4,000 in PBS-T) and goat anti-rabbit IgG (1:6,000 in PBS-T), both conjugated with horseradish peroxidase (Sigma Aldrich), were added to wells containing standard sera and anti-cholera toxin, respectively. Finally, 100 µL of substrate solution (phosphate-citrate 0.1 M; pH 4.0) containing 0.4 mg/mL o-phenylenediamine and 0.1% (v/v) H_2_O_2_ were added to each well and incubated for 15 min at room temperature in the dark. The plates were measured for its absorbance at 450 nm in plate reader spectrophotometer (Thermo, Plate).

### Mice and guinea pig vaccination

Female Swiss Webster mice, 6 to 8 weeks old, were randomly segregated into 5 groups of 10 animals each. Group one was comprised of animals vaccinated with 50 µg of rLTB/H_C_C/H _C_D without conventional adjuvant to evaluate the potential of rLTB as a humoral immune response adjuvant. Group two was comprised of animals vaccinated with 50 µg of rLTB/H_C_C/H _C_D plus a 15% aluminum hydroxide suspension (Al(OH)_3_). Group three was comprised of animals vaccinated with the same molar ratio of rH _C_C/H _C_D plus 15% Al(OH)_3_. Group four was comprised of animals vaccinated with a commercial toxoid vaccine; the dose used was one-twentieth of the dose recommended for cattle. Group five (control group) was comprised of animals vaccinated with 200 µL sterile PBS. Three doses were administered intraperitoneally on days 0, 14 and 28. Blood samples were collected on day 35 from the retro-orbital plexus, and sera were separated by centrifugation (7 min; 3,000 *x g*), pooled and frozen until further use.

Guinea pigs were vaccinated subcutaneously on days 0 and 21 with 200 µg of each chimera. The groups, each with 10 randomly sorted animals, were the same as those described above for the experiments in mice. Blood samples were collected directly from the heart on day 42, and sera were separated as previously described and used for a mouse neutralization bioassay.

### Evaluation of the humoral immune response

The humoral immune responses of the mice and guinea pigs were evaluated by a mouse neutralization bioassay performed in accordance with the Brazilian Ministry of Agriculture, Livestock and Food Supply in its ministerial directive n. 23 [28]. Briefly, 1 L+ of each standardized native toxin was incubated for 60 min at 37 °C with 1 mL of undiluted and diluted (1:2, 1:3, 1:5, 1:10 and 1:20) pooled sera containing neutralizing antibodies. 1 L+ is the minimal dose of toxin that, when incubated with 1 IU/mL of standard antiserum, is still able to kill the whole mice population. After the incubation period, two mice, weighting 18 to 22 g, were inoculated intravenously and observed for 72 h, with survival checked every 24 h. The titers, expressed in international units per mL (IU/mL), were directly calculated as the minor dilution of serum that, even after incubation with 1L+ of BoNT, is still capable of killing the inoculated mice.

### Statistical analyses

Statistix9 (Analytical Software) was used to perform ANOVA and Tukey’s test to analyze antigenicity and establish significant differences in the ELISA results.

## Results

### Vector construction and the expression and purification of recombinant chimeras

**Figure 2:**
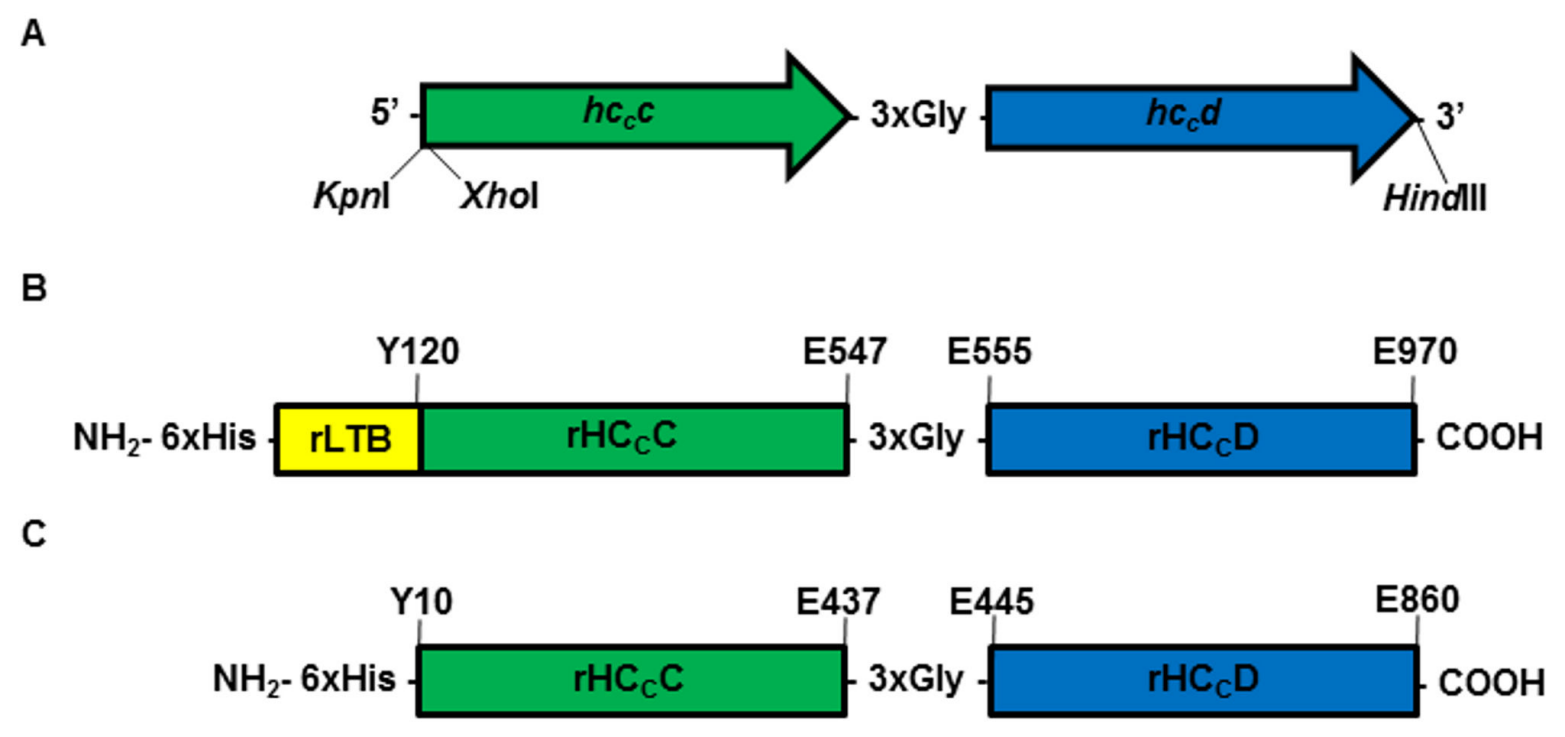
Schematic representations of the constructed gene and fusion proteins. (A) Structure of the gene designed to encode the fusion antigens. The *h_c_c* (green) is flanked by *Kpn*I and *Xho*I sites, respectively, at the 5’ end. The coding sequence of *h_c_d* (blue) is linked at the 3’ end of *h_c_c* by three Glycine codons and is flanked by *Hind*III at the 3’ end. (B) Representation of the recombinant chimera region, followed by the adjuvant rLTB connected to rH _C_C, a three-Glycine linker (3xGly) and the rH _C_D at the C-terminal region. (C) Representation of the recombinant chimera rH _C_C/rH _C_D. The resulting protein has the same characteristics as shown in B, except for the absence of the rLTB sequence connected to the N-terminal region of HC _C_C.

**Figure 3:**
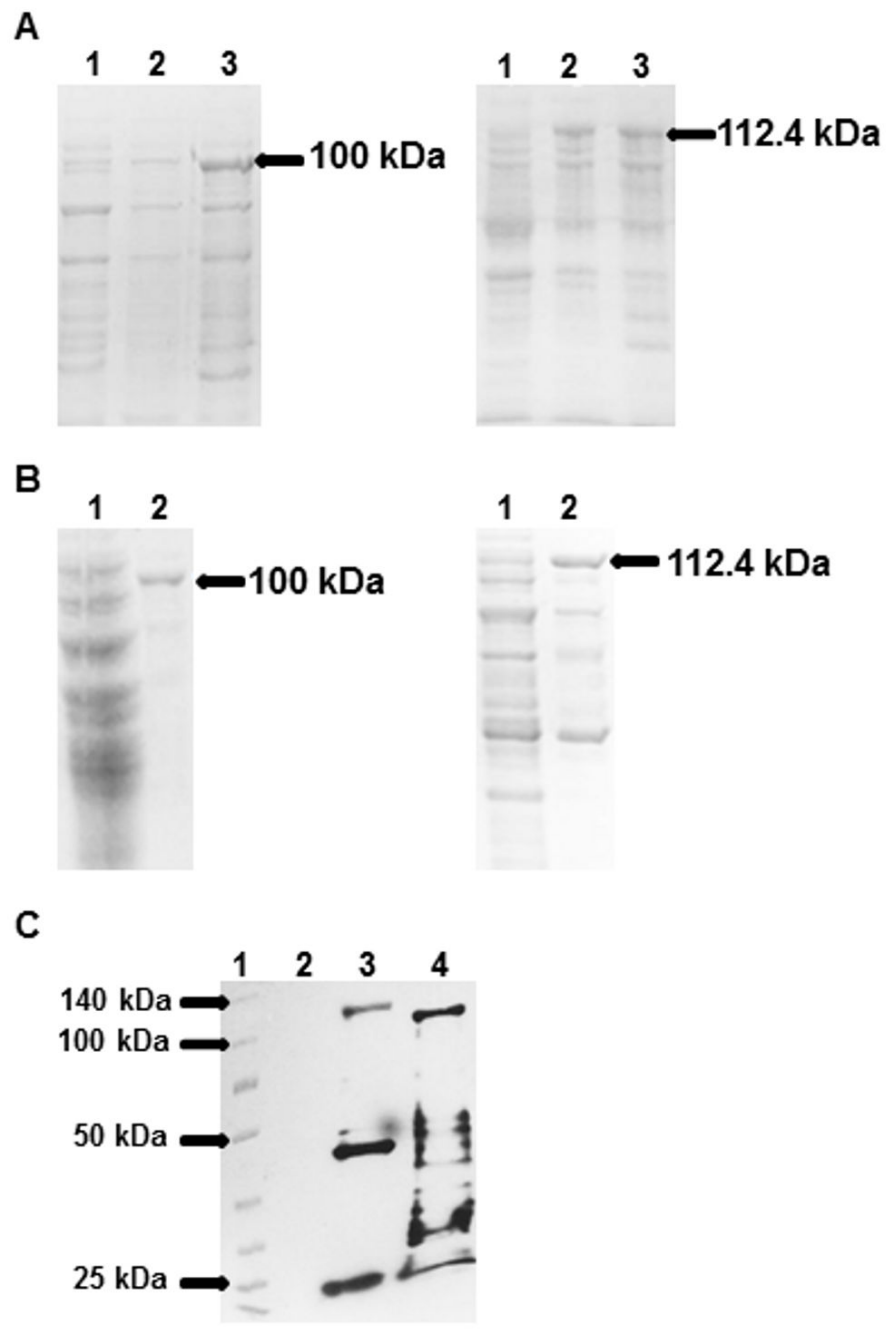
SDS-PAGE and Western blot analysis of protein expression and purification. (A) SDS-PAGE of the whole *E. coli* cell extract after 3 h of induction of both constructs. Left, 1: Untransformed *E. coli* BL21 (DE3) Star; 2: *E. coli* BL21 (DE3) Star transformed with pAE/*h*_*c*_ *c-h *_*c*_ *d* before induction; 3: *E. coli* BL21 (DE3) Star transformed with pAE/*h*_*c*_ *c-h *_*c*_ *d* after induction. Right, 1: Untransformed *E. coli*BL21 (DE3) Star; 2: *E. coli* BL21 (DE3) Star transformed with pAE/*ltb-h *_*c*_ *c-h *_*c*_ *d* before induction; 3: *E. coli* BL21 (DE3) Star transformed with pAE/*ltb-h *_*c*_ *c-h *_*c*_ *d* after induction. (B) SDS-PAGE to evaluate the purity and integrity of purified proteins after Ni-affinity chromatography. Left, rH _C_C. Right, rH _C_D. All lanes designated “1” are untransformed *E. coli* BL21 (DE3) Star, and the lanes designated “2” are the purified antigens. (C) Anti-his Western blot to characterize the purified proteins. 1: Spectra Multicolor Broad Range Protein Ladder (Thermo Scientific), 2: Untransformed *E. coli* BL21 (DE3) Star, 3: rLTB/H _C_C/H _C_D, 4: rH _C_C/H _C_D.

The synthetic gene encoding for H_C_C and H_C_D (Fig. 2A), cloned by Epoch Biolabs Inc. (USA) into the pUC19 cloning vector, was digested with endonucleases to release a fragment encoding both chimeras together (Figure 2B and 2C). These genes were cloned into either the pAE/*ltb* or pAE expression vectors, resulting in two different constructs: pAE/*ltb-h *_*c*_ *c-h *_*c*_ *d* and pAE/*h*_*c*_ *c-h *_*c*_ *d*. Digestion with endonucleases confirmed the correct cloning of each construct. Thereafter, *E. coli* BL21 (DE3) Star transformed with pAE/*ltb-h *_*c*_ *c-h *_*c*_ *d* or pAE/*h*_*c*_ *c-h *_*c*_ *d* expressed recombinant proteins of approximately 112 and 100 kDa, respectively, which were the expected molecular weights; however, additional bands with lower molecular weights than expected were also present (Figure 3A and 3B). The additional bands are likely to be truncated proteins because they all reacted with anti-polyHistidine in a Western blot analysis (Figure 3C). Both chimeras were obtained as inclusion bodies and were efficiently solubilized in lysis buffer containing 0.4% and 0.2% NLS, respectively. The one-step refolding process using PBS with 0.05% Triton X-100 did not interfere with protein solubility, since aggregates were not observed. Using this process, it was possible to achieve up to 100 mg of purified recombinant antigen per liter of culture.

### Recombinant chimeras are recognized by standard sera

**Figure 4:**
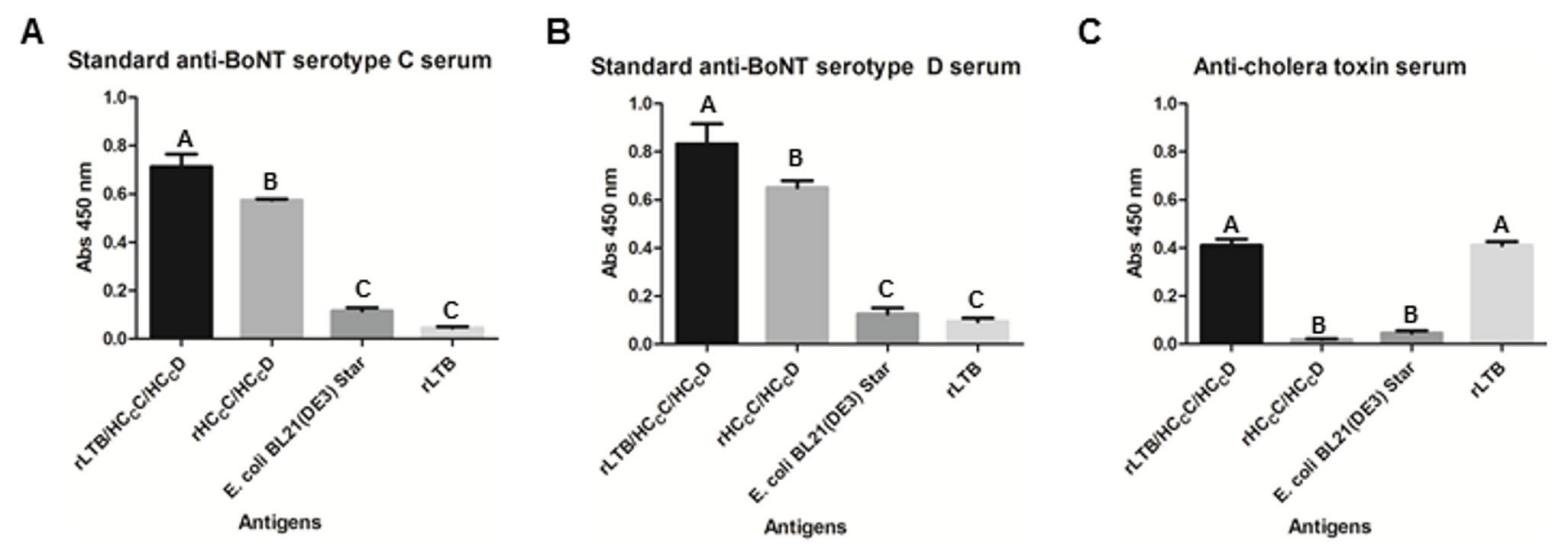
Antigenicity evaluation of the recombinant chimeras by ELISA using standard anti-BoNT C and D sera. Anti-BoNT C serum (A) showed that the rLTB/H _C_C/H _C_D antigen has more antigenic epitopes than rH _C_C/H _C_D, while anti-BoNT D serum (B) shows no difference between these two antigens. Anti-cholera toxin serum (C) was used to evaluate the antigenicity of the rLTB domain. Crude protein extract from *E. coli* BL21 (DE3) Star and purified rLTB were used as controls for the specificity of the three sera. Different letters above the bars (A, B, or C) indicate significant differences (p < 0.001) according to Tukey’s test. Absorbances (Abs) shown were measured at 450 nm wavelength.

Recombinant chimeras were characterized with ELISAs using standard sera containing anti-BoNTs serotypes C and D and anti-cholera toxin. The ELISAs showed positive reactions of anti-BoNT/C, anti-BoNT/D and anti-CT against rLTB/H_C_C/H _C_D. As expected, rH _C_C/H _C_D did not react only with anti-CT. Furthermore, the negative control (crude protein extract of *E. coli* BL21 (DE3) Star) did not react with any sera. Additionally, the standard sera anti-BoNT/C and anti-BoNT/D did not react with rLTB (Figure 4). These results indicate that all domains of the recombinant chimeras are antigenic because they were recognized by standard sera and were significantly different from the negative controls (p < 0.001).

### Recombinant chimeras are innocuous and elicit neutralizing antitoxins

Neither mice nor guinea pigs vaccinated with recombinant chimeras presented abnormal responses or lesions at their vaccination sites, indicating the safety of both recombinant chimeras. Sera samples collected from vaccinated animals were used to evaluate the humoral immune response. The mouse neutralization bioassay results from both mice and guinea pigs are presented in Table 2. In both experiments, rLTB/H_C_C/H _C_D plus Al(OH)_3_ resulted in the best vaccination strategy. Pooled sera from the mice contained 2 IU/mL antibodies against both C and D toxins. Pooled sera from the guinea pigs contained 5 IU/mL and 10 IU/mL antibodies against toxins C and D, respectively. In mice, the other constructs elicited less than 2 and 1 IU/mL antibodies against C and D, respectively. Similarly, in guinea pigs, the other antigens elicited less than 1 IU/mL antibodies against both toxins. The commercial vaccine tested in mice was only effective against BoNT serotype C, whereas in guinea pigs, it elicited the expected antibody levels for both serotypes. The vaccination of guinea pigs with rLTB/H_C_C/H _C_D plus Al(OH)_3_ met the requirements of the Brazilian Ministry of Agriculture, Livestock and Food Supply in its ministerial directive N^°^.23 [28] and induced at least two times more antitoxins against BoNT serotype D than the commercial vaccine.

**Table 2:** Levels of neutralizing antibodies against BoNTs serotypes C and D in vaccinated mice and guinea pigs.

Vaccine formulation	Mice^a^		Guinea pigs^a^	
	Serotype C	Serotype D	Serotype C	Serotype D
rLTB/H_C_C/H_C_D	2 IU/mL	1 IU/mL	≤ 1 IU/mL	≤ 1 IU/mL
rLTB/H_C_C/H_C_D + Al(OH)_3_	2 IU/mL	2 IU/mL	5 IU/mL	10 IU/mL
rH_C_C/H_C_D + Al(OH)_3_	2 IU/mL	1 IU/mL	≤ 1 IU/mL	≤ 1 IU/mL
Commercial vaccine	5 IU/mL	1 IU/mL	5 IU/mL	3 IU/mL
PBS	ND^b^	ND^b^	ND^b^	ND^b^

^a^ Values obtained by mouse neutralization bioassay.

^b^ ND, not detectable.

The formulations with only rLTB or Al(OH)_3_ as single adjuvants induced low levels of neutralizing antibodies. In contrast, vaccination using both rLTB and Al(OH)_3_ induced high levels of antitoxins. Clearly, there is a synergism between rLTB and Al(OH)_3_, which is important for the induction of a stronger humoral immune response against the two analyzed BoNTs.

## Discussion

Botulism in cattle has been the cause of great economic losses in the past years [5,7,8], with reports indicating that it is a potential source of food-borne botulism in humans [9,10]. This disease is therefore considered a widespread problem for both livestock production and human health. Currently, toxoids used to vaccinate animals are produced by *C. botulinum* fermentation and the further inactivation of produced toxins by formaldehyde. These procedures result in a non-predictable process that involves high biological risks because the native BoNTs are the most potent toxins known to humans [29]. Additionally, residual formaldehyde may affect the vaccine’s safety [17]. The strategy utilized by our group overcomes these problems because heterologous protein expression in *E. coli* can be highly regulated, and the H_C_ domain of the BoNTs is non-toxic, thus eliminating risks to workers during vaccine production.

We developed a bivalent recombinant vaccine with only one fermentative and downstream process, thereby facilitating vaccine production by avoiding additional fermentation steps. Most studies using *E. coli* to produce recombinant H_C_ domains yield an average of 40 mg/L, except when a production optimization procedure is performed [30]. When only H_C_C and H_C_D production is considered, the yield average is higher, reaching 70 mg/L-culture [13,18,31,32]. The expression strategy we employed was able to produce up to 100 mg of recombinant protein per liter of culture, indicating high expression efficiency. Thus, considering the previously reported expression yield for individual H_C_s, our chimeras exhibit a more effective production system for vaccines, with only one bioprocess needed to produce what would normally require at least 4 different fermentation steps. An attempt to optimize the expression of rLTB/H_C_C/H _C_D using the same parameters as described by Yarí et al. [33] in modified M9 medium did not interfere with the protein yield or the protein solubility (data not shown).

Our mouse and guinea pig vaccination results demonstrated that both fused constructs did not cause adverse side effects and are capable of inducing neutralizing antibodies (Table 2). These results were expected because the H_C_ domains of BoNTs are non-toxic and capable of inducing protection against botulism [18]. Although the fusion of H_C_C, H_C_D and LTB might eliminate a few important protective epitopes, the immune responses of the mice and guinea pigs suggest that some epitopes were still present, thereby allowing the generation of immunity to botulism. Although a linker consisting on 3xGly was added between rH _C_C and rH _C_D, it is possible that the conformational epitopes of rH _C_C could not fold properly when compared to rH _C_D, which is flanked only by rH _C_C. This is possible because H_C_D elicited higher neutralization titers than H_C_C in guinea pigs. Three glycine residues were chosen because this amino acid has no side chain and thus confers flexibility between the domains, allowing the correct protein folding of each one. In fact, previous studies have described the effective use of glycine-rich linkers, such as (Gly _4_Ser)_3_ and (Gly–Ser–Gly)_2_, even in vaccine studies due to the flexibility of glycine [34–39]. These linkers are larger than the 3xGly used in this work, supporting the hypothesis that it is too short to allow the proper folding of H_C_C, and thus explaining the difference in the production of antibodies against toxins C and D in guinea pigs. Sakamoto et al. [38] described the influence of linker length and flexibility upon functionality of each domain of a recombinant chimera. Thus, it is possible that using a (Gly _4_Ser)_3_ linker instead of only 3xGly may result in a stronger humoral immune response.

Despite different vaccination schedules, a remarkable discrepancy was observed between the results of the two animal models. While the chimeric vaccine induced high neutralizing antibodies levels in guinea pigs, the mice did not show a similar result. Although neutralization assays are not commonly performed with mouse sera, our data are not in accordance with the literature, as previous studies have shown that BALB/c and ICR mice can generate high levels of protection against several BoNTs [18,40]. One possible explanation for these experimental differences is the mouse strain that we used (Swiss Webster). This type of mouse is genetically heterogeneous and thus has the advantage of representing a varied population. In addition, it is not an enhanced T_H_2 responder strain, such as the BALB/c strain, which could explain the low antibody production against the antigens that were used. In contrast, guinea pigs are ideal model organisms for immunology tests against botulism and, thus, it is expected that high levels of immunological responses could be generated [41,42].

The rLTB/H_C_C/H _C_D chimera plus Al(OH)_3_, induced high levels of neutralizing antibodies against both serotypes in guinea pigs. It is noteworthy that the Brazilian Ministry of Agriculture, Livestock and Food Supply determined that a vaccine against bovine botulism must induce minimum of 5 IU/mL and 2 IU/mL of anti-BoNT C and D neutralizing antibodies, respectively, to be approved [28]. In our results from the mouse neutralization bioassay, the chimera rLTB/H_C_C/H _C_D plus Al(OH)_3_ induced 5 IU/mL and 10 IU/mL for serotypes C and D, respectively, which is in accordance with the government’s mandate. These values were comparable to the commercial vaccine and much higher than the other tested constructs. Takeda et al. [43] vaccinated ducks with a C/D mosaic toxoid (the H_C_ portion consisted of serotype D) and obtained an average of 6 IU/mL, as determined by the mouse neutralization bioassay. Moreover, in our study, sera samples were obtained from guinea pigs three weeks after the last vaccination dose, in a manner similar to that of Takeda [43]. Nonetheless, we obtained an average of 7.5 IU/mL when considering both serotypes, which highlights the potential of our chimera. However, guinea pigs vaccinated with either rLTB/H_C_C/H _C_D alone or rH _C_C/H _C_D plus Al(OH)_3_ did not display an appropriate humoral immune response.

The best results were obtained using the constructs containing LTB, a powerful adjuvant of humoral immune response [22,23,25,26]. The presence of LTB was essential for the development of a strong humoral immune response when it acted in synergism with Al(OH)_3_. Thus far, there have not been any works reporting a bivalent vaccine against botulism using a single polypeptide chain containing antigens and adjuvant. Additionally, there have not been any works evaluating LTB as an adjuvant in vaccines for preventing botulism. Conceição et al. [22] used LTB fused to the R1 antigen of *Mycoplasma* *hyopneumoniae* P97 adhesin to immunize mice without conventional adjuvants. This treatment induced the production of strong humoral and cellular immune responses, highlighting the use of LTB as an adjuvant of the immune response with antigens fused to its C-terminal portion. Indeed, the fusion of other antigens to the C-terminal end of LTB does not impair its biological activity, as was already reported [44,45].

Zeng et al. [46] published a similar work with a trivalent recombinant chimera against *C. perfringens* toxins, in which vaccines with fused toxins or co-administered antigens were demonstrated to have higher immunogenicity than antigens alone, eliciting a high titer of neutralizing antibodies. These results corroborate ours, in which synergism between LTB and Al(OH)_3_, together with vaccination with fused antigens, induced elevated titers of neutralizing antibodies. The large difference between the levels of neutralizing antibodies generated by vaccination of guinea pigs with Al(OH)_3_ and either rLTB/H_C_C/H _C_D or rH _C_C/H _C_D support these findings and indicate that LTB indeed works as a systemic humoral immune response adjuvant, even though it was originally described to be a mucosal adjuvant [47]. Additionally, animals vaccinated with rLTB/H_C_C/H _C_D without conventional adjuvant developed low levels of neutralizing antibodies, suggesting that synergism between Al(OH)_3_ and LTB is essential for the induction of an appropriate humoral immune response.

Both LTB and Al(OH)_3_ are capable of inducing T_H_2-type responses [26,48], which in turn activate B cells through secretion of IL-4 and IL-5 [49], resulting in B cell proliferation and a strong humoral response consisting of the production of both s-IgA and IgG [50], as well as other isotypes. Aluminum hydroxide also creates a deposit of antigens, which extends the antigen exposure time to the immune cells, resulting in a stronger and more specific systemic response. Although the mechanism of action of LTB remains unclear, it has been shown that its activity stems from its ability to bind to GM1 ganglioside receptors [26,47], which are present on almost all mammalian cells, including antigen-presenting cells [51]. In this manner, LTB plays a key role by easing antigen uptake and presentation by dendritic cells, macrophages and, most importantly, B cells. Furthermore, LTB has been shown to up-regulate the expression of essential molecules for the development of an appropriate and strong immune response, such as the B7.1 and B7.2 co-stimulatory molecules. It also augments the expression of chemokine receptors and MHC class II on antigen-presenting cells [52]. Consequently, the adjuvant effects induced by both LTB and Al(OH)_3_ act synergistically and complement each other, resulting in a stronger systemic humoral response. Thus, the rLTB/H_C_C/H _C_D molecule can be considered a “3 in 1” product, as it contains (1) a vaccine against cattle botulism serotype C, (2) a vaccine against cattle botulism serotype D, and (3) an adjuvant molecule, all in a single polypeptide chain.

In conclusion, we describe in this study the potential of the recombinant chimera rLTB/H_C_C/H _C_D as a novel strategy to prevent botulism in cattle by vaccination. The use of rLTB/H_C_C/H _C_D plus Al(OH)_3_ could be considered a potential commercial product, although large-scale production must still be established. We also corroborated the results from other studies [22,25] establishing the potential of LTB as a humoral immune response adjuvant. Furthermore, our study also provides insights for studies with respect to other bacterial toxins.
